# The Effect of Sepsis on Myocardial Function: A Review of Pathophysiology, Diagnostic Criteria, and Treatment

**DOI:** 10.7759/cureus.26178

**Published:** 2022-06-21

**Authors:** Nabeeha Khalid, Pragnesh D Patel, Rahmah Alghareeb, Afshan Hussain, Marvi V Maheshwari

**Affiliations:** 1 Cardiology, Omar Hospital and Cardiac Centre, Lahore, PAK; 2 Research, St. George's University School of Medicine, St. George's, GRD; 3 College of Medicine, University of Baghdad, Baghdad, IRQ; 4 Research, Dow Medical College and Dr. Ruth K. M. Pfau Civil Hospital Karachi, Karachi, PAK; 5 Research, Our Lady of Fatima University College of Medicine, Valenzuela, PHL

**Keywords:** echocardiography, infection, myocardial dysfunction, septic shock, cardiomyopathy, sepsis

## Abstract

Sepsis remains a worldwide challenge for physicians with many patients admitted to ICUs with septic shock. Septic shock management involves targeted treatment to control infections, reduce end-organ damage, and reverse the injury. Sepsis-induced myocardial dysfunction or septic cardiomyopathy remains an avenue to be explored with regard to underlying pathophysiology and definite treatment guidelines. This article has compiled various studies to explain the possible mechanisms involved in the development of septic cardiomyopathy and the existing diagnostic criteria including radiological and laboratory tests to assess septic cardiomyopathy. Furthermore, the article highlights management options currently available for physicians dealing with myocardial dysfunction secondary to sepsis.

## Introduction and background

Sepsis is a dysregulated host response to infection leading to end-organ damage. Sepsis is the leading cause of ICU-related deaths worldwide [1]. With the emergence of antimicrobial resistance, sepsis represents a worldwide health concern. With the increase in the aging population, the incidence of sepsis is increasing as well. According to the Centers for Disease Control and Prevention (CDC), in 2011, sepsis accounted for over $20 billion of total hospital costs in the USA [2]. The core of sepsis research is inflammation, and it is now clear that sepsis involves the activation of both pro-inflammatory and anti-inflammatory responses along with immunological changes [3]. When sepsis worsens, it progresses to multi-organ involvement and the development of septic shock, which presents as hypotension and perfusion abnormalities, eventually leading to death. Septic shock is a subset of sepsis characterized by abnormalities in circulation and metabolism and carries a high risk of mortality [2]. The current management of sepsis remains supportive care. There is room for further pathological exploration and therapeutic interventions to advance in the management of this condition.

In adults with septic shock, ventricular dysfunction has been observed in 60% of patients in the first three days, and global heart function is affected [4]. A hospital-based study revealed that 43% of patients with bacteremia had positive troponin indicating myocardial damage [5]. Sepsis-induced cardiomyopathy (cardiac systolic function impairment) carries a risk of 80% mortality as opposed to sepsis with normal systolic function [6].

The underlying pathophysiology of septic shock secondary to severe sepsis is based on myocardial contractile dysfunction, which causes acute heart failure with low or normal diastolic pressures [7]. This hemodynamic impairment is functional secondary to microcirculatory dysfunction and autonomic dysregulation [8] and metabolic due to inflammatory cytokines, dysregulation of inflammatory cells, and metabolism of nitric oxide [9]. The early stages of septic shock are characterized by a hyperdynamic phase in which there is increased cardiac output, reduction in systemic vascular resistance, and tachycardia [8]. The late-stage and more common clinical presentation is a hyperdynamic phase in which there is a reduction in left ventricular (LV) ejection fraction, dilatation of the left ventricle, and hypotension [10].

There is no treatment for septic cardiomyopathy; however, it is a reversible condition, and research targets earlier identification of myocardial dysfunction before changes in ejection fraction. Although various pathological mechanisms have been theorized, the underlying mechanism of dysfunction remains unclear, and further investigations are warranted. This article aims to explore the various underlying pathological mechanisms involved in the development of myocardial dysfunction in sepsis. It also touches on the correlation between pathological mechanisms and clinical manifestations of septic cardiomyopathy as well as management modalities to prevent cardiac involvement and improve mortality in sepsis.

## Review

Discussion

Septic cardiomyopathy is myocardial involvement in sepsis and is defined as myocardial depression that is reversible and resolves in 7-10 days. A postmortem necropsy study carried out by Ammann et al. in 2001 revealed myocardial injury in more than half of patients with sepsis [11].

Pathophysiology

The pathophysiology of septic cardiomyopathy is complex, and various mechanisms have been investigated to contribute to the development of myocardial dysfunction in sepsis.

Bacterial endotoxin is one principal mediator of LV dysfunction. In a 1989 study carried out by Suffredini et al., nine subjects were given an intravenous bolus of endotoxin (*Escherichia coli*) against a control group of six subjects who were given intravenous saline, and left ventricular ejection fraction was measured. The study concluded that endotoxin causes depression in the left ventricular ejection fraction [12].

Cytokines and inflammatory mediators have a direct inhibitory effect on myocardial contractility, leading to myocardial dysfunction. In a randomized trial carried out by Husebye et al. in 2014, the levels of various inflammatory markers were measured in STEMI patients with heart failure after infusion of levosimendan versus placebo. The study concluded that high circulating levels of interleukin (IL)-8 were associated with less improvement of left ventricular function after six weeks, suggesting a possible role of IL-8 in reperfusion-related myocardial injuries [13]. A retrospective observational study carried out by Chen et al. in 2021 at a single center aimed to evaluate the association between complements C3 and C4, lymphocyte markers CD4 and CD8, and interleukins IL-1β, IL-2R, IL-6, IL-10, and IL-8 in patients with established sepsis-induced myocardial dysfunction. The study concluded that patients with myocardial dysfunction had significantly elevated levels of IL-8 [14]. An animal trial carried out by Eichenholz et al. in 1992 studied the effects of transfusing human recombinant tumor necrosis factor (TNF), and the resulting cardiovascular abnormalities were studied for 10 days. The study concluded that the group receiving TNF infusion had decreased left ventricular ejection fraction as compared to the control group [15].

Nitric oxide also plays a significant role in myocardial dysfunction in sepsis. Nitric oxide is produced by cardiac cells and has a crucial role in cardiac performance. Endothelial nitric oxide synthase (eNOS/NOS3) is responsible for the production of NO, which in normal hemodynamics has a positive ionotropic and lusitropic effect, while it is also responsible for metabolism and contractility in myocardial cells. While nitric oxide has a positive ionotropic effect, this is only at lower concentrations. As the concentration increases, nitric oxide has a negative ionotropic effect. High levels of nitric oxide during sepsis induce myocardial dysfunction. In an animal study carried out by van de Sandt et al. in 2013, cardiac functions were assessed in septic wild-type mice with endogenous NOS versus the control group. The study concluded that NOS3 contributed to increased bioactive nitric oxide, leading to myocardial dysfunction [16].

The imbalance of oxidative status leading to the overproduction of reactive oxygen species (ROS) is a major contributor to myocardial injury. Free radicals are molecules with additional electrons, causing them to be highly reactive and toxic. Oxygen-containing free radicals are termed reactive oxygen species. ROS plays many important roles in the life cycle of cells including the induction of cell signaling pathway, intracellular secondary messenger activation, and cell defense mechanism. However, sepsis causes increased production of these free radicals, which cannot be countered by anti-oxidative mechanisms. These ROS lead to oxidative damage to proteins, lipids, and DNA, leading to mitochondrial damage and apoptosis and promoting inflammation [17]. Oxidative stress can lead to the activation of the nuclear enzyme polyadenosine 5′-diphosphate (ADP)-ribose polymerase (PARP), which leads to decreased myocardial contractility. The overactivation of PARP leads to the depletion of its substrate NAD+, which impairs ATP formation and eventually contributes to cardiac cell death. PARP also modulates inflammatory pathways. In a prospective and observational study conducted in the intensive care units of a university hospital by Soriano et al., 25 patients with sepsis were followed. The study concluded that there was PARP activation in the hearts of septic patients with impaired cardiac function [18].

ROS also leads to the inhibition of oxidative phosphorylation in mitochondria and thus depletes the cell of ATP. This state of non-utilization of oxygen is termed cytopathic hypoxia. Various other mechanisms have been identified for mitochondrial damage in sepsis, including ischemia and hypoxia. All these mechanisms lead to the increased permeability of the mitochondrial membrane, leading to an influx of ions into the mitochondrial matrix and subsequent rupture of mitochondria, releasing proapoptotic mediators into the cell, leading to the activation of apoptosis in cardiac myocytes. This results in the disruption of the contractile apparatus of the myocyte (actin/myosin) and loss of cardiac myocytes [19,20].

**Figure 1:**
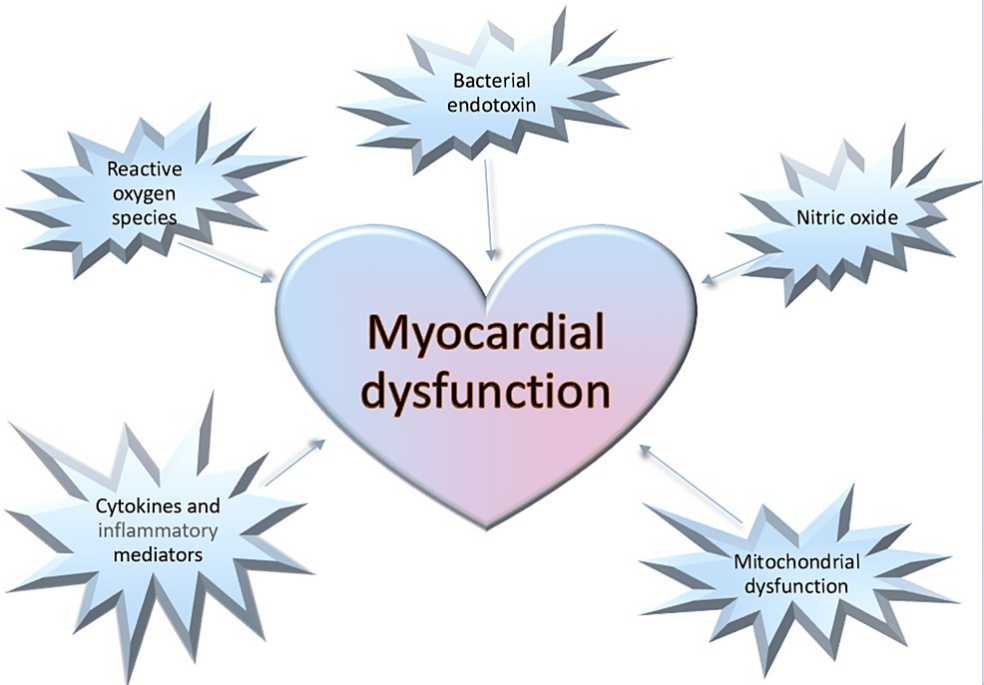
Pathological mechanisms leading to septic cardiomyopathy Image credits: Nabeeha Khalid

To date, many studies have been conducted, but the current data is still not sufficient to identify the exact mechanism of the development of septic cardiomyopathy. However, many underlying mechanisms have been theorized (Figure 1), and there appears to be an underlying correlation hinting at multiple pathological mechanisms leading to cardiomyopathy in sepsis.

Diagnosis

Septic cardiomyopathy is defined by a reversible decrease in left ventricular ejection fraction. Although echocardiographic findings in septic cardiomyopathy also include isolated diastolic dysfunction and RV systolic dysfunction, this review article will focus on LV systolic dysfunction. Echocardiography is currently the investigation of choice for the diagnosis of sepsis-induced myocardial dysfunction. Echocardiography aims to assess changes in the LV cavity and changes in LV systolic dysfunction. Systolic function measured as left ventricular ejection fraction is the first parameter described in the diagnosis of septic cardiomyopathy [21]. Bedside echocardiography in patients with sepsis and septic shock is a quick and useful modality to guide management in patients.

The key to therapeutic success in septic shock is early recognition of hemodynamic compromise, and echocardiography is an excellent tool for both diagnoses and monitoring of patients with sepsis. In a retrospective study carried out by Lan et al. in 2019, 3,291 patients who were admitted with sepsis from 2001 to 2012 at Beth Israel Deaconess Medical Center were studied. The study concluded that the utilization of echocardiography in patients with septic shock led to improved 28-day outcomes [22]. Similarly, a prospective study carried out by Şahin et al. in 2017 analyzed 140 children admitted to the pediatric intensive care unit from March 2013 to August 2013. The study concluded that echocardiography is an invaluable tool for management [23]. In a study carried out by Fu et al. in 2022, patients admitted to the ICU with sepsis were identified as those who had received echocardiography versus those who had not. The study concluded that in critically ill patients, the use of echocardiography was associated with better 28-day outcomes [24]. The observational study carried out by Brown et al. in 2012 reviewed 78 patients admitted to the ICU. The study concluded that grade II and grade III LV diastolic dysfunction were associated with higher mortality, indicating inadequate fluid resuscitation and hence highlighting the significance of echocardiography in the management of septic shock [25]. In a randomized control trial by El-Nawawy et al. in 2017, 90 children admitted to the pediatric intensive care unit were divided into a study group monitored with serial echocardiography and a control group. The trial concluded that serial echocardiography allowed early recognition of septic cardiomyopathy and hypovolemia, leading to timely management resulting in shock reversal time reduction [26]. The importance of echocardiography in the management of septic shock has been summarized in Table 1.

**Table 1:** Significance of echocardiography in the management of septic shock ICU: intensive care unit

Reference and author	Design	Number of cases	Population	Conclusion
Lan et al. (2019) [22]	Retrospective observational study	3,291	Patients admitted with sepsis to the ICU	Patients admitted to the ICU who underwent echocardiography had a better 28-day outcome.
El-Nawawy et al. 2017 [26]	Randomized controlled trial	90	Patients admitted to the pediatric ICU	Serial echocardiography provided crucial data for early recognition of septic myocardial dysfunction, reducing shock reversal time.
Şahin et al. 2017 [23]	Observational study	140	Age 45 days-18 years admitted to the pediatric ICU	Echocardiography is an invaluable tool for the management of critically ill children.
Brown et al. 2012 [25]	Observational study	78	Patients admitted to the ICU	Grade II and grade III diastolic dysfunction were associated with greater mortality due to inadequate fluid resuscitation, suggesting the role of echocardiography in the management of shock.
Fu et al. 2022 [24]	Retrospective observational study	13,844	Patients admitted to the ICU	The use of transthoracic echocardiography leads to a better 28-day outcome.

The measurement of the global longitudinal strain (GLS) via speckled echocardiography has recently emerged and is now considered more accurate in the diagnosis of myocardial dysfunction. Speckle tracking uses an automated computer algorithm that tracks movements in regions selected by the user throughout the cardiac cycle. The program allows the assessment of changes in contractility and thus LV function. Strain imaging detects changes in LV systolic function before changes in left ventricular ejection fraction, thus allowing earlier detection of myocardial dysfunction [27]. In a 2014 systemic review carried out by Kalam et al., the global longitudinal strain had a superior prognostic value in detecting major cardiac events than the left ventricular ejection fraction [28]. In a case-control study carried out by Ng et al. in 2016 on a study group of patients with septic shock as compared to a control group with sepsis, echocardiography and speckle tracking were performed. The study concluded that speckle echocardiography could detect significant changes in left ventricular function not detected by conventional echocardiography band and could also demonstrate the reversible nature of cardiomyopathy in sepsis [29].

Cardiac biomarkers are also used as a tool for the diagnosis of cardiac involvement in sepsis. Troponin I is a highly sensitive marker for myocardial damage, but it can be elevated without evidence of myocardial ischemia. In a prospective cohort study carried out by Lim et al. in 2005, 115 patients admitted to the ICU were enrolled and were categorized into those who had elevated troponin and myocardial infarction (MI) versus those who did not have MI. The study concluded that 47% of critically ill patients had elevated troponin, but only 26% had MI [30]. Elevated cardiac biomarkers can be used to assess myocardial involvement in patients with sepsis. In a prospective case-control study carried out by Ammann et al. in 2001, 20 patients with sepsis and systemic inflammatory response (SIRS) were compared to a control group without coronary artery disease or myocarditis. The study found that cardiac troponin I was elevated in 85% of patients with sepsis and septic shock [11].

B-type natriuretic peptide (BNP) is a hormone produced by the heart in response to changes in pressure inside the heart. NT-proBNP is an inactive prohormone of BNP. The levels of BNP and NT-proBNP are elevated in heart failure and have been used in the prognosis of congestive heart failure and ischemic heart disease. In a 2007 observational cohort study by Varpula et al., the predictive value of NT-proBNP in critically ill patients was evaluated. The study results showed that NT-proBNP values were increased in patients with sepsis and septic shock [31]. In a 2005 study by Brueckmann et al., 57 patients with sepsis had BNP levels measured. The results of the study suggested that the levels of NT-proBNP are very likely related to cardiovascular dysfunction [32].

Management

The standard treatment for sepsis focuses on infection control and hemodynamic optimization with fluid resuscitation and vasopressors. The treatment of sepsis-induced myocardial dysfunction is the same strategy. Since the dysfunction is reversible, therapies to effectively manage sepsis will lead to the resolution of cardiomyopathy [33]. The key guidelines for surviving sepsis campaign suggested that dobutamine infusion can be added to vasopressin in the case of myocardial dysfunction suggested by elevated cardiac filling pressures and decreased cardiac output [34]. Dobutamine administration in septic shock leads to markedly increased cardiac index as described in the study by Vincent et al. in 1990 [35]. However, despite dobutamine increasing cardiac index in septic patients, it does not improve overall mortality. In a randomized trial carried out by Hayes et al. in 1994, the treatment group was given dobutamine versus the control group. The trial concluded that despite achieving adequate cardiac index and systemic oxygen delivery, there were no improvements in the outcome [36].

The beta-blockade has been suggested to reduce heart rate and decrease mortality in sepsis. In a randomized control trial by Morelli et al. in 2013, 77 patients with septic shock were given intravenous esmolol to reduce heart rate below 95 bpm. The study showed improved heart rates without any adverse clinical outcomes; however, mortality reduction still needed further investigation [37].

Levosimendan is a positive ionotropic drug and increases the effect of calcium on myofilaments. Despite being ionotropic, it does not cause increased heart rate and therefore is predicted to have better outcomes in patients as compared to dobutamine. In a 2015 meta-analysis by Zangrillo et al., randomized trials of levosimendan use in septic shock were included with mortality being the primary outcome. The study concluded that levosimendan was associated with improved mortality in patients with septic shock as compared to standard ionotropic therapy [38]. However, data on the use of levosimendan in established septic cardiomyopathy is still limited.

In the case of nonresponsiveness to vasopressin and fluid resuscitation, an intra-aortic balloon pump (IABP) can be used to increase cardiac output. It can also be used to reduce the dose of vasopressors. Intra-aortic balloon pump is a mechanical circulatory support device. The IABP assists the heart by reducing afterload, leading to increased organ perfusion and coronary blood flow. An animal trial carried out by Solomon et al. in 2009 to study the effect of intra-aortic balloon counter pulsations in mechanically ventilated canine models concluded that IABC prolongs survival and reduces the need for vasopressors [39]. While IABP is used in patients with cardiac shock secondary to various cardiac conditions including myocardial infarction, its use in septic cardiomyopathy is rare. It is considered a last resort in nonresponsive septic shock rather than standard therapy.

**Figure 2:**
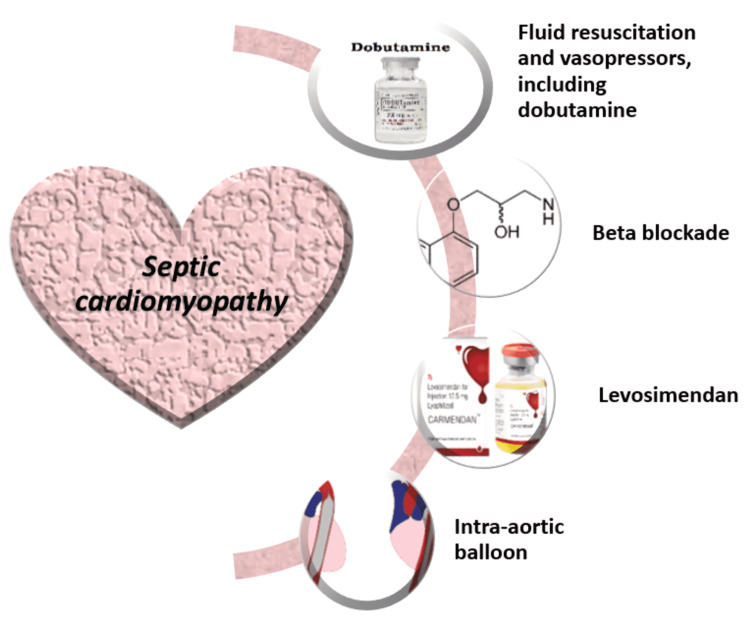
Suggested treatment options for septic cardiomyopathy Image credits: Nabeeha Khalid

Therefore, the management options for septic cardiomyopathy remain limited and focus primarily on treating the underlying shock augmented with fluid and vasopressor therapy to maintain cardiac output and hemodynamics. Figure 2 summarizes the treatment options for septic cardiomyopathy.

This review article covers the basic pathological mechanisms suggested to lead to cardiomyopathy in sepsis and the diagnostic and prognostic markers. It also touches on the management options for septic cardiomyopathy, but as data for this is insufficient, the article is limited to the management of sepsis at large. Septic cardiomyopathy also encompasses isolated LV diastolic dysfunction and RV systolic dysfunction, which is still a debate, and thus, this article is limited to the effect of sepsis on left ventricular systolic dysfunction. There is a need for further investigation to determine the definite cause of myocardial dysfunction and novel treatment guidelines.

## Conclusions

Septic cardiomyopathy is a reversible decrease in myocardial contractility secondary to the release of bacterial endotoxin, increased inflammatory markers, increased reactive oxygen species, nitric oxide, and mitochondrial dysregulation. Septic cardiomyopathy is characterized by the dilation of the LV cavity, decreased left ventricular ejection fraction, and return to baseline LV function within 7-10 days. Septic cardiomyopathy is diagnosed by clinical signs of sepsis including low cardiac output and the development of septic shock. The investigation of choice is echocardiography to assess for changes in LV systolic function and guide management. Speckle echocardiography for the measurement of the global longitudinal strain is a novel investigation for the early detection of myocardial involvement in sepsis. Cardiac markers including high sensitivity troponin and BNP can be used to diagnose and assess prognosis in patients with nonischemic myocardial damage secondary to sepsis.

The management of septic cardiomyopathy targets the management of underlying sepsis and achieving hemodynamic stability using fluid and vasopressors. The challenge is the lack of definite guidelines targeting the treatment of septic cardiomyopathy. Although a large number of studies are present on septic cardiomyopathy, there is still room for further investigation to determine the definite pathology of myocardial dysfunction and definite guidelines directed toward treating septic cardiomyopathy to improve outcomes in patients with sepsis and reduce mortality.
